# fNIRS-based Neurorobotic Interface for gait rehabilitation

**DOI:** 10.1186/s12984-018-0346-2

**Published:** 2018-02-05

**Authors:** Rayyan Azam Khan, Noman Naseer, Nauman Khalid Qureshi, Farzan Majeed Noori, Hammad Nazeer, Muhammad Umer Khan

**Affiliations:** 1 Department of Mechatronics Engineering, Air University, Islamabad, Pakistan; 2 Department of Biomedical Engineering, Faculty of Electronic Information and Electrical Engineering, Dalian University of Technology, Dalian, China; 3 Department of Electrical and Computer Engineering, Institute of Systems and Robotics, University of Coimbra, Coimbra, Portugal

**Keywords:** Functional near-infrared spectroscopy, Brain-computer interface, Primary motor cortex, Hemodynamic response filter, Linear discriminant analysis, Support vector machine, Computed torque controller

## Abstract

**Background:**

In this paper, a novel functional near-infrared spectroscopy (fNIRS)-based brain-computer interface (BCI) framework for control of prosthetic legs and rehabilitation of patients suffering from locomotive disorders is presented.

**Methods:**

fNIRS signals are used to initiate and stop the gait cycle, while a nonlinear proportional derivative computed torque controller (PD-CTC) with gravity compensation is used to control the torques of hip and knee joints for minimization of position error. In the present study, the brain signals of walking intention and rest tasks were acquired from the left hemisphere’s primary motor cortex for nine subjects. Thereafter, for removal of motion artifacts and physiological noises, the performances of six different filters (i.e. Kalman, Wiener, Gaussian, hemodynamic response filter (hrf), Band-pass, finite impulse response) were evaluated. Then, six different features were extracted from oxygenated hemoglobin signals, and their different combinations were used for classification. Also, the classification performances of five different classifiers (i.e. k-Nearest Neighbour, quadratic discriminant analysis, linear discriminant analysis (LDA), Naïve Bayes, support vector machine (SVM)) were tested.

**Results:**

The classification accuracies obtained from SVM using the hrf were significantly higher (*p* < 0.01) than those of the other classifier/ filter combinations. Those accuracies were 77.5, 72.5, 68.3, 74.2, 73.3, 80.8, 65, 76.7, and 86.7% for the nine subjects, respectively.

**Conclusion:**

The control commands generated using the classifiers initiated and stopped the gait cycle of the prosthetic leg, the knee and hip torques of which were controlled using the PD-CTC to minimize the position error. The proposed scheme can be effectively used for neurofeedback training and rehabilitation of lower-limb amputees and paralyzed patients.

## Background

Neurological disability due specifically to stroke or spinal cord injury can profoundly affect the social life of paralyzed patients [1–3]. The resultant gait impairment is a large contributor to ambulatory dysfunction [4]. In order to regain complete functional independence, physical rehabilitation remains the mainstay option, owing to the significant expense of health care and the redundancy of therapy sessions. Such devices are developed as alternatives to traditional, expensive and time-consuming exercises in busy daily life. In the past, similar training sessions on treadmills performed using robotic mechanisms have shown better functional outcomes [1, 2, 5–7]. However, these devices have limitations particular to given research and clinical settings. Therefore, wearable upper- and lower-limb robotic devices have been developed [7, 8], which are used to assist users by actuating joints to partial or complete movement using brain intentions, according to individual-patient needs.

**Fig. 1:**
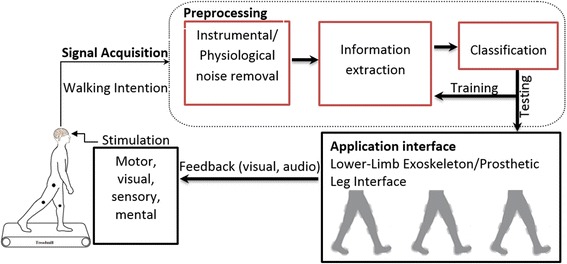
Schematic of BCI

To date, various noninvasive modalities including functional magnetic resonance imaging (fMRI), electroencephalography (EEG) and functional near-infrared spectroscopy (fNIRS) have been used to acquire brain signals. fNIRS is a relatively new modality that detects brain intention with reference to changes in hemodynamic response. Its fewer artifacts, better spatial resolution and acceptable temporal resolution make it the choice for comprehensive and promising results in, for example, rehabilitation and mental task applications [9–20]. The main brain-computer interface (BCI) challenge in this regard is to extract useful information from raw brain signals for control-command generation [21–23]. Acquired signals are processed in the following four stages: preprocessing, feature extraction, classification, and command generation. In preprocessing, physiological and instrumental artifacts and noises are removed [24, 25]. After this filtration stage, feature extraction proceeds in order to gather useful information. Then, the extracted features are classified using different classifiers. Finally, the trained classifier is used to generate control commands based on a trained model [23]. Figure 1 shows a schematic of a BCI.

Previous studies on signal-acquisition techniques have shown promising outcomes, but rehabilitation applications require the best possible results [3, 4, 26]. In Eliana et al. [27], a treadmill was used to acquire EEG-based walking brain signals for sensorimotor applications with 87% accuracy. In Andreea et al. [28], EEG-based walking-intention signals were detected for stroke patients with an accuracy of 82%. Their data indicated that patients highly motivated for rehabilitation-related tasks tended to have higher success rates. In Naseer et al. [29], two-class motor imagery movements were analyzed using an LDA classifier. With their employed modality, fNIRS, the best features were found to be signal mean (SM) and signal slope (SS). By reducing the task period to between 2 and 7 s, the accuracies were improved to 77.56 and 87.28%, respectively. In Rea et al. [30], lower-limb movement for gait rehabilitation was detected based on fNIRS signals. They were able to acquire fNIRS signals in their chronic stroke patients during preparation for hip movement with 67.77 ± 11.35% accuracy. In Zhao et al. [31], a prosthetic controller was proposed for a bipedal robot. A walking gait pattern was found for the robot mechanism while an online optimized trans-femoral prosthesis control method (i.e. control Lyapunov function (CLF)-based quadratic programs (QPs) with variable impedance control) was tested on the knee and ankle joints of the prosthetic device. Azimi et al. [32] proposed stable robust adaptive impedance control for a prosthetic limb. A regressor-based nonlinear robust model was designed with reference to an adaptive impedance controller. In Richter et al. [33], dynamic modeling and simulation-based control of a prosthesis were performed, focusing on two-degree-of-freedom robot modeling, parametric estimation and feedback control for mimicking of hip motions. Perrey [34] explored neural gait control using fNIRS, specifically looking at the relevant cortical areas. In Venkatakrishnan [35], meanwhile, examined and discussed a rehabilitation-based brain machine interface (BMI) application for stoke patients.

The previous literature on the subject of rehabilitation shows that classification accuracy in the online setting is compromised by, among other problems, false triggering. Therefore, we also present a method to ensure that a correct command is always sent to a prosthetic leg (details are given in Section 3.1.1).

In this study, we acquired fNIRS walking signals of healthy subjects. Raw signals might contain noises and artifacts that can be removed using adaptive or band-pass filtering [25, 36]. In order to avoid such noises, the following six filters were compared for signal processing: Kalman, Wiener, finite impulse response (FIR), hemodynamic response (hrf), Band-pass, and Gaussian. Five classifiers, namely quadratic discriminant analysis (QDA), linear discriminant analysis (LDA), support vector machine (SVM), k-Nearest Neighbour (KNN), and Naïve Bayes (NB), were analyzed for acquisition of maximum classification accuracies. For offline BCI, SVM showed greater statistical significance (*p* < 0.01) as compared with the other classifiers; however, in consideration of execution delay and minimum computation cost, for online BCI, we used LDA with combinations of six features: SS, SM, signal peak (SP), signal kurtosis (KR), signal skewness (SK), and signal variance (SV). Walking intention was then used to initiate and stop the gait cycle of the proposed prosthetic leg model. For minimization of discomfort, a nonlinear computed torque controller (CTC) with gravity compensation was applied to two active joints in the hip and knee and one passive joint in the ankle for position control and reduction of error in waking patterns [37–39]. Given its effective simulation of classical limb-type and mobile robotics, the Peter Corke® robotics tool box was used to minimize position error [40]. The proposed system is applicable not only to paralyzed patients but also, and with little modification, to amputees and elderly people.

## Method

### Experimental protocol

In this study we used dynamic near-infrared optical tomography (DYNOT; NIRx Medical Technologies, NY, USA). DYNOT operates on two wavelengths, 760 and 830 nm. The machine sampling frequency used for signal acquisition was 1.81 Hz. Prior to the experimentation, verbal consent was obtained from all of the subjects. Nine healthy male members having normal or corrected-to-normal vision were recruited for the study. All were right-handed and of 30 ± 3 median age. As discussed in the literature, the best region in which to acquire fNIRS-based BCI signals for self-paced walking is the primary motor cortex (M1); thus, signals were acquired from the M1 in the left hemisphere [34, 41–43]. The participants had no history of motor disability or any visual, neurological disorder. All of the experiments were performed in accordance with latest Declaration of Helsinki.

### Experimental paradigm

**Fig. 2:**
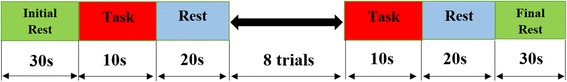
Experimental paradigm

In accordance with the literature [22, 41], the subjects were asked to take a rest for 30s in a quiet room before the start of each experiment. The experimental paradigm consisted of 10s walking on a treadmill followed by 20s rest while standing on the treadmill. All of the subjects started their walk with the right leg. For each subject, 10 trials were performed, and a 30s rest was given at the end of each experiment for baseline correction of the signals. Excluding the initial and final rest, the total length of each experiment was 300 s for each subject. Self-paced walking, which is to say, according to each subject’s comfort level, was performed. Figure 2 shows the experimental paradigm.

### Experimental configuration

**Fig. 3:**
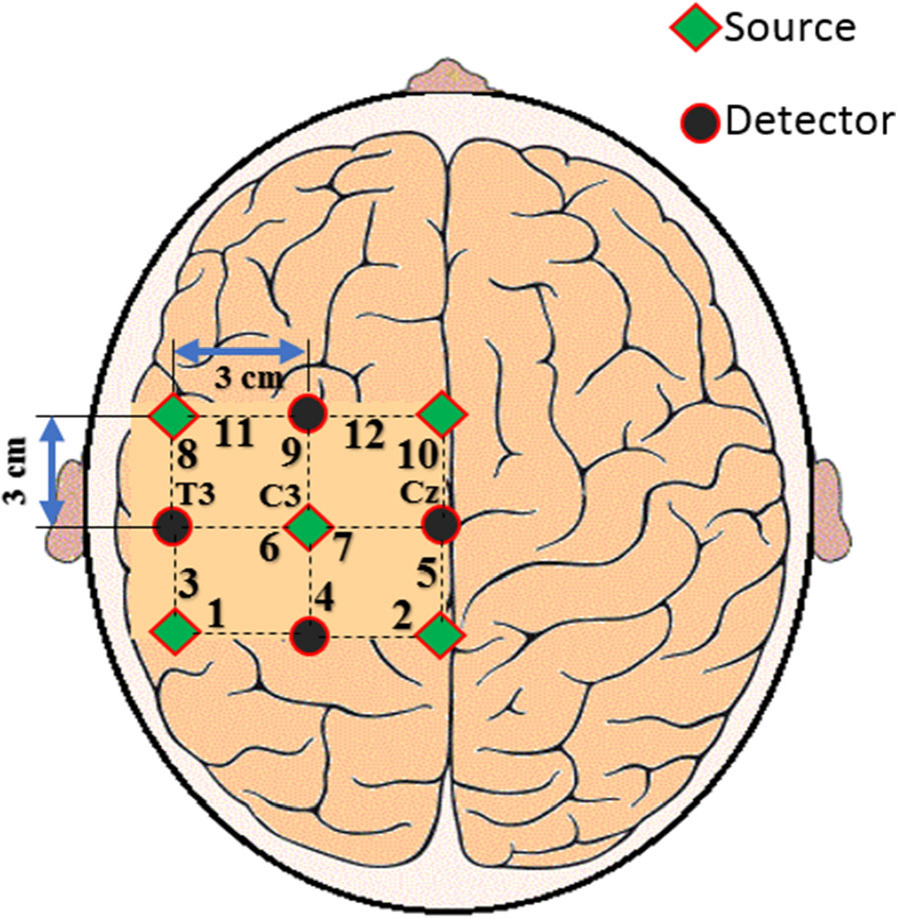
Optode placement with channel configuration on left hemisphere of motor cortex [82]. T3, C3, and Cz are reference points in the international 10-20 system

To acquire fNIRS-based walking brain signals, 9 optodes were placed on the left hemisphere of the M1, among which 4 were Near Infrared (NI) light detectors and 5 were sources. Twelve (12) channels were formed as per the defined configuration, and a 3 cm distance was maintained between a source and a detector. The source/detector configuration with channels is shown in Fig. 3.

#### Signal acquisition

The Modified Beer-Lambert Law (MBLL) was used to convert raw optical density signals into oxy- and deoxy-hemoglobin concentration changes (*∆c*_HbO_(*t*) and *∆c*_HbR_(*t*)) [18, 44].

1 $$ \left[\frac{\varDelta {c}_{HbO}(t)}{\varDelta {c}_{HbR}(t)}\right]=\frac{{\left[\begin{array}{cc}{\alpha}_{HbO}\left({\lambda}_1\right)& {\alpha}_{HbR}\left({\lambda}_1\right)\\ {}{\alpha}_{HbO}\left({\lambda}_2\right)& {\alpha}_{HbR}\left({\lambda}_2\right)\end{array}\right]}^{-1}\left[\begin{array}{c}\varDelta A\left(t,{\lambda}_1\right)\\ {}\varDelta A\left(t,{\lambda}_2\right)\end{array}\right]}{d\ast l} $$

Where *l* is the source and detector distance, *d* is the curved path length factor, *A*(*t*, *λ*_1_), *A*(*t*, *λ*_2_) is the absorption at two different instants, *α*_*HbR*_(*λ*), *α*_*HbO*_(*λ*) are the extinction coefficient of HbO and HbR in [μM^−1^ cm^−1^], and *Δc*_*HbR*_(*t*), *Δc*_*HbO*_(*t*) are the concentration changes of HbR and HbO in [μM].

### Signal processing

**Fig. 4:**
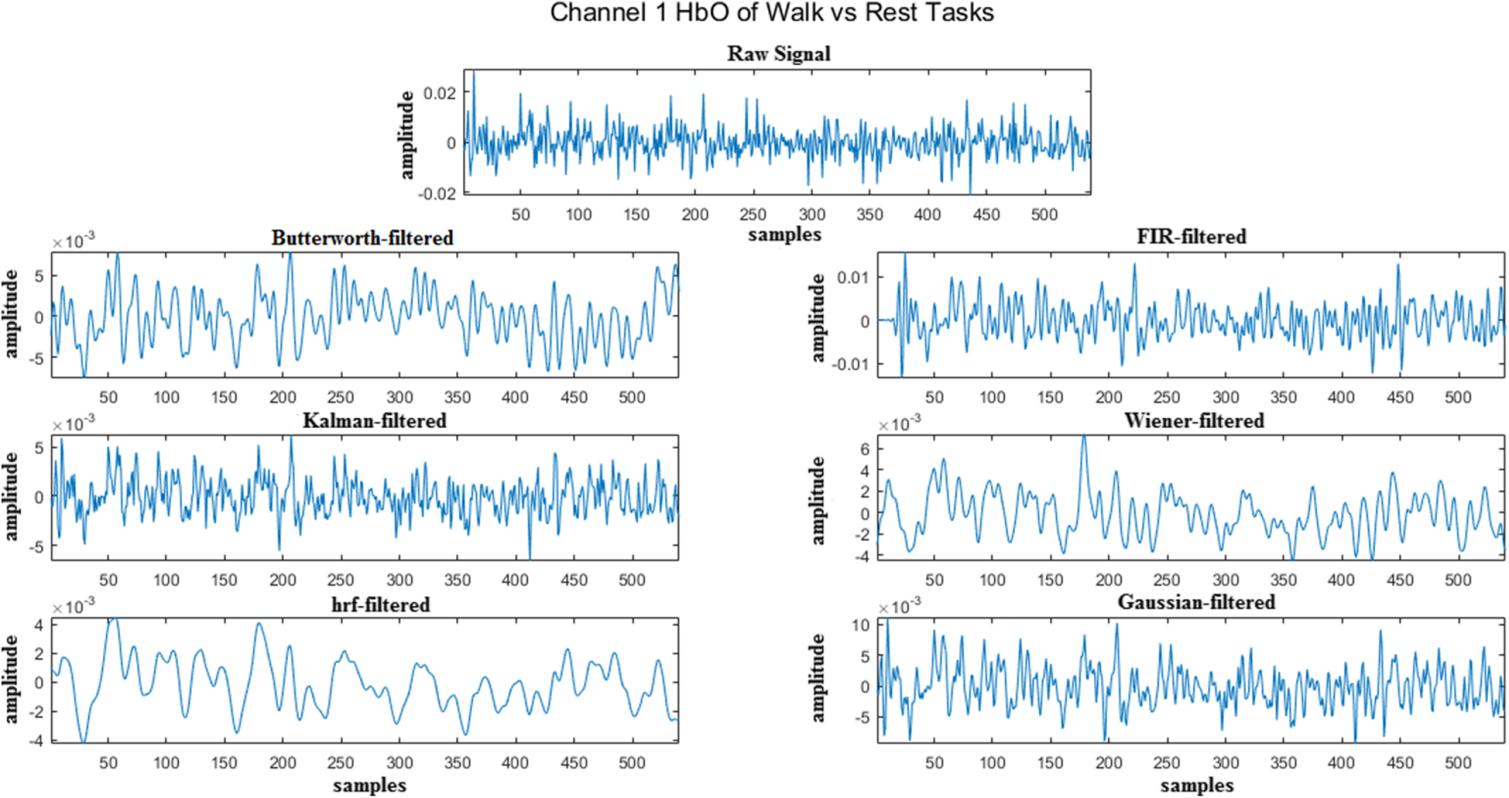
The filtered HbO signals of channel 1 for subject 1 using all six filters

The brain signals acquired were filtered using different filters to attain maximum accuracy. To eliminate high- and low-frequency physiological or instrumental noises such as heartbeat (1-1.5 Hz), respiration (~ 0.5 Hz), artifacts, blood pressure (Mayer waves), and others, signals were filtered with a low-pass filter having cut-off frequency of 0.5 Hz and a high-pass filter having cut-off frequency of 0.01 Hz, in accordance with the literature [23]. The employed filters were Butterworth, Finite Impulse Response (FIR), Kalman, Wiener, hemodynamic response (hrf) and Gaussian. Butterworth and FIR filters were 4th order. Kalman filter with a discrete model was implemented [45], whereas time-varying Wiener filter, based on short-time Fourier series was implemented as in [46]. Gaussian and hrf filters were applied using NIRS-SPM toolbox developed by [17]. These filters consider Gaussian kernel and canonical hemodynamic response function, respectively, for smoothing of the time-series signal. Figure 4 shows the filtered HbO signals of channel 1 for subject 1 using all six filters.

### Feature extraction

In this study, six different features were extracted using spatial average of all 12 measured channel [47]. Six statistical properties (SM, SK, KR, SS, SP, SV) of the averaged signal were calculated for the entire task and rest sessions. For SM, the calculation was as follows:

2 $$ SM=\frac{1}{N}{\sum}_{i=1}^N{Z}_i $$

where *N* is the total number of observations and *Z*_*i*_ represents the *Δc*_*HbO*_(*t*) across each observation. SK was calculated according to the asymmetry of the signal values around the mean relative to a normal distribution:

3 $$ skew(Z)=E\left[{\left(\frac{Z-\mu }{\sigma}\right)}^3\right] $$

where *σ* is the standard deviation of *Z* and *E* is expected value of *Z*. KR was calculated as:

4 $$ kurt(Z)=E\left[{\left(\frac{Z-\mu }{\sigma}\right)}^4\right] $$

**Fig. 5:**
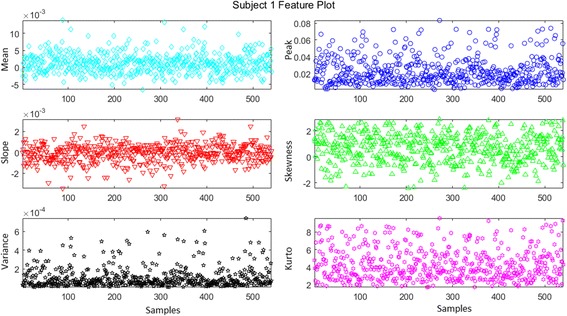
Scatter plot of features for subject 1

SS was calculated by using the *polyfit* function in MATLAB®, which fits a line to all data points. To calculate SP, the *max* function in MATLAB® was used. The features are rescaled between 0 and 1 using the equation

5 $$ {x}^{\prime }=\frac{x-\min (x)}{\max (x)-\min (x)} $$

where *x*′ is the rescaled feature, *x* ∈ *R*^*n*^denotes the original feature values, min *x* is the smallest value, and max *x* is the largest value. Figure 5 provides the scatter plot of subject 1 for all features.

### Classification

#### SVM

SVM is used for offline BCI classification. Due to its non-linear nature moreover, it is widely employed to achieve high classification performance [48–51]. Thus, by using SVM, high-dimensional data can be scaled and errors can be explicitly controlled. In order to attain the maximum classification accuracy, SVM creates hyperplanes to maximize the margins between the classes. The vectors known as hyperplanes are named support vectors [23, 48–52].

The optimal solution r* is obtained by minimizing the following cost function between a hyperplane and the nearest training data points.

Minimize

6 $$ \kern0.5em \frac{1}{2}\ {\left\Vert w\right\Vert}^2+C{\sum}_{i=1}^n{\upxi}_i $$

Subject to

7 $$ \kern0.75em {y}_i{\left({w}^T\ {x}_i+b\right)}^3\ge 1-{\upxi}_i,\kern2.5em {\upxi}_i\ge 0 $$

where *w*^*T*^, *x*_*i*_ ϵ *R*^2^and *b* ϵ *R*^1^, ‖*w*‖^2^ = *w*^T^*w*, *C* is the trade-off parameter between the margin and the error, ξ_*i*_ is the measured training data, and *y*_*i*_ is the class label for the i^th^ sample. We used a third-degree polynomial kernel function with *C* = 0.5. 10-fold cross-validation was then applied for estimation of classification accuracies.

#### LDA

LDA is the most common classifier used for pattern recognition in BCI offline and online systems, due to its low computational cost and high-speed performance. To separate classes from each other, LDA finds the projection to a line so that the two classes are well separated [47, 53]. LDA’s main objective is to perform dimensionality reduction, for which it minimizes the variance within each projected class and maximizes the distance between the means of projected classes.

This is done by maximizing the Fisher’s criterion given below:

8 $$ J(v)=\frac{v^T{S}_bv\ }{v^T{S}_wv} $$

where *S*_*b*_ and *S*_*w*_ are the between-class and within-class scatter matrices defined as

9 $$ {S}_{\mathrm{b}}={\left({m}_1-{m}_2\right)\left({m}_1-{m}_2\right)}^{\mathrm{T}},\kern0.5em {S}_{\mathrm{w}}=\sum_{x_n\epsilon 1}\left({x}_n-{m}_1\right){\left({x}_n-{m}_2\right)}^{\mathrm{T}}+\sum_{x_n\epsilon 2}\left({x}_n-{m}_1\right){\left({x}_n-{m}_2\right)}^{\mathrm{T}}\kern3.5em $$

where *m*_1_ and *m*_2_ represent the group means of classes C_1_ and C_2_, respectively, and *x*_*n*_ denotes the samples. A vector *v* that satisfies (9) can be reformulated, as a generalized eigenvalue problem, as.

10 $$ {S}_{\mathrm{w}}^{-1}{S}_{\mathrm{b}}v=\uplambda v $$

The optimal *v* is the eigenvector corresponding to the largest eigenvalue of${S}_{\mathrm{w}}^{-1}{S}_{\mathrm{b}}$, or it can be written as.

11 $$ v={S}_{\mathrm{w}}^{-1}\left({m}_1-{m}_2\right) $$

provided that *S*_w_ is non-singular. The 10-fold cross-validation was applied for estimation of classification performance.

#### KNN

KNN predicts the test sample’s category in accordance with the k training samples that are nearest neighbors to test sample and classifies it based upon largest category probability [54]. Assume there are *j* training categories as (*C*_1_, *C*_2_, …, *C*_*j*_), class *Y* is the feature vector of all training samples, *E*_*i*_ is one of the neighbor in the training set, *X*(*E*_*i*_, *C*_*j*_) ∈ {0, 1} indicate whether *E*_*i*_ belongs to class *C*_*j*_, and *Sim*(*Y*, *E*_*i*_) is the similarity function for feature data *Y* and *E*_*i*_, then the probability density function *P*(*Y*, *C*_*j*_) for *Y* and *C*_*j*_ is given as [54]:

12 $$ P\left(Y,{C}_j\right)=\sum \limits_{E_i\in KNN} Sim\left(Y,{E}_i\right)\cdotp X\left({E}_i,{C}_j\right) $$

where, *Sim*(*Y*, *E*_*i*_) was calculated using the Euclidean distance methods. For closest training data of class, the parameter k was considered 1 while 10-fold cross-validation was performed for estimation of accuracies.

#### QDA

QDA maximizes and minimizes ratio of between-class and within-class variance, provided observations are normally distributed for each class *i*, the ratio test can be performed by [54]:

13 $$ \frac{f_i(X)}{f_j(X)}=\frac{\frac{1}{2\pi {\left|{\sum}_i\right|}^{\raisebox{1ex}{$1$}\!\left/ \!\raisebox{-1ex}{$2$}\right.}}{\mathit{\exp}}^{\left[-\frac{1}{2}{\left(X-{\mu}_i\right)}^T{\sum}_i^{-1}\left(X-{\mu}_i\right)\right]}}{\frac{1}{2\pi {\left|{\sum}_j\right|}^{\raisebox{1ex}{$1$}\!\left/ \!\raisebox{-1ex}{$2$}\right.}}{\mathit{\exp}}^{\left[-\frac{1}{2}{\left(X-{\mu}_j\right)}^T{\sum}_j^{-1}\left(X-{\mu}_j\right)\right]}}\kern0.5em <t $$

for some threshold t. Where, *X* is the feature vector, *μ*_*i*_, *μ*_*j*_ are the normally distributed mean and ∑_*i*_, ∑_*j*_ are the covariance matrix of class *i*, *j*. After rearrangement the separating quadratic surface between classes can be obtained.

#### NB

NB is considered among commonly used classifiers for classification that is based on probabilistic approach. The model used for NB is as follows [55]:

14 $$ P\left(k|y\right)=\frac{P\left(y|k\right)\ P(k)}{P(y)} $$

where *P*(*k*| *y*) is the class feature probability for a specified feature, *P*(*y*| *k*) is the given class probability of feature, *P*(*y*) is the feature prior probability and *P*(*k*) is the class prior probability.

### Kinematic model of prosthetic leg

**Fig. 6:**
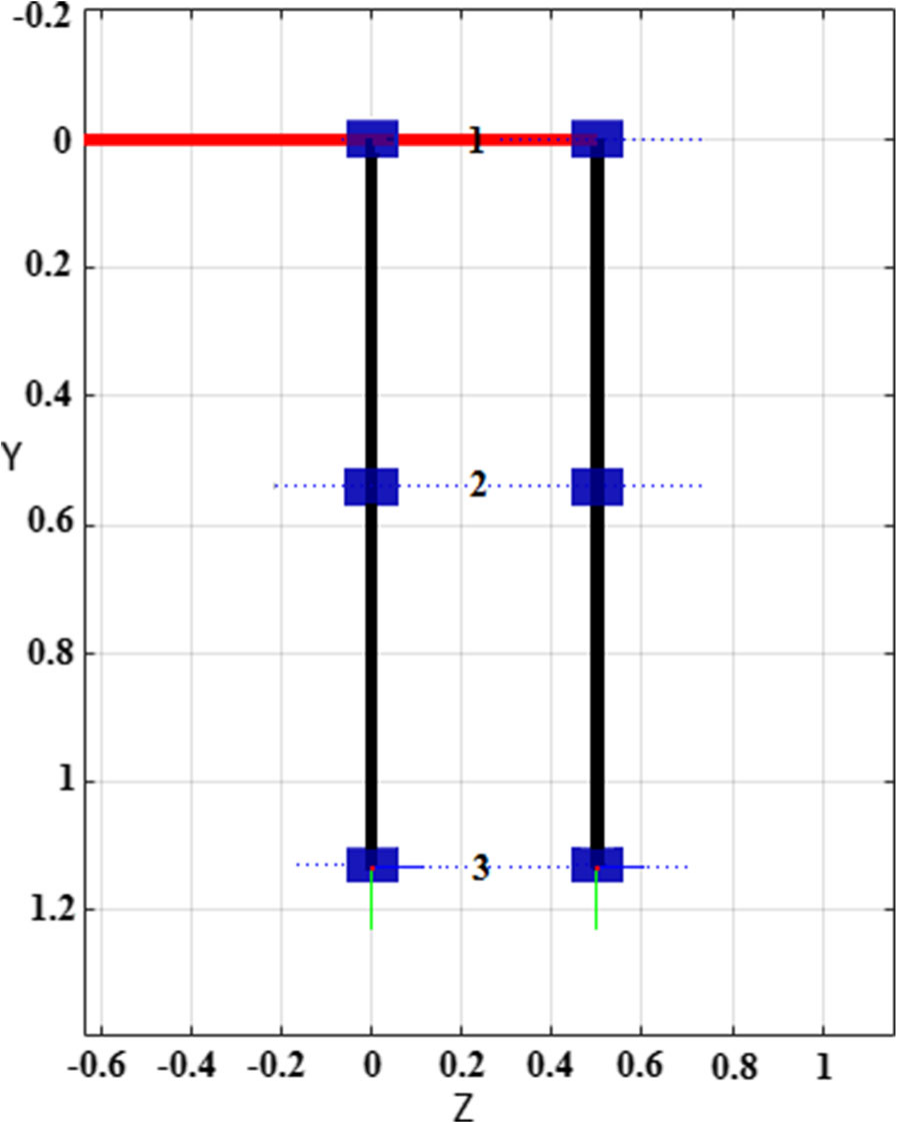
Front view of biped robot

A human leg includes hip, knee and ankle joints. The most efficient joint is the knee, which has to bear the entire body’s weight [56]. The knee and hip joints are the key joints used in locomotion; therefore, the proposed model is kept simple by considering only the hip and knee joints for articulation and the ankle joint as fixed. Therefore, only 2 degrees of freedom (DOF) were considered: 1 DOF for the hip joint and 1 for the knee [39, 57–62]. Moreover, the base was assumed fixed, making it two serial-link manipulators in which one manipulator is moved 180° out of phase with the other one [61–64]. The average thigh clearance given in the literature for a man is 0.78 in, and for women, 0.90 in [65, 66]. The end-effector position and orientation were derived from the Denavit-Hartenberg (D-H) notation [57, 59, 67, 68]. The front view of the proposed model is shown in Fig. 6, and the leg parameters are listed in Table 1.

**Table 1:** Prosthetic-leg D-H notations [68]

Link no.	*α*_*i* − 1_	*α*_*i* − 1_	*d*_*i*_	*θ*_*i*_
1.	0	L_1_	0	*θ*_1_
2.	0	L_2_	0	*θ*_2_
3.	0	L_3_	0	*θ*_3_

#### Prosthetic leg parameters

The length parameters of the prosthetic leg are provided in Table 2.

**Table 2:** Prosthetic leg parameters [95]

Parameters	Link	Length (m)
L_1_	Thigh	0.2
L_2_	Shank	0.2
L_3_	Pelvic	0.03

#### Dynamic model of prosthetic leg

The dynamics of an n-link robotic leg can be expressed by the following set of n equations [68]

15 $$ M\ddot{q}+b\dot{q}+g=\tau $$

where q is an n-dimensional vector describing the joint positions of the robot, τ is the vector of input torques, *g* is the gravitational torque, *b* represents the Coriolis and centripetal forces caused by the motion of the link, and *M* is the nxn inertia matrix of the robot.

The coordinates for the hip and knee joints become [69]

16 $$ {x}_1={x}_a+{r}_1\sin {\theta}_1 $$

17 $$ {y}_1={y}_a-{r}_1\cos {\theta}_1 $$

18 $$ {x}_2={x}_a+{L}_1\sin {\theta}_1+{r}_2\sin {\theta}_2 $$

19 $$ {y}_2={y}_a-{L}_1\cos {\theta}_1-{r}_2\cos {\theta}_2 $$

Considering the kinetic and potential energy of the entire system, the Langrangian becomes [69]

20 $$ \mathrm{L}=\frac{1}{2}{m}_1\left({\dot{x}}_1^2+{\dot{y}}_1^2\right)+\frac{1}{2}{I}_1{\dot{\theta}}_1^2+\frac{1}{2}{m}_2\left({\dot{x}}_2^2+{\dot{y}}_2^2\right)+\frac{1}{2}{I}_2{\dot{\theta}}_2^2-{m}_1{y}_1g-{m}_2{y}_2g $$

Substituting the joint coordinates and solving the Jacobian matrix, which is the differential relationship between the joint displacements and the end-effecter position, we obtain the hip and knee joint torques [69, 70] as

21 $$ {\tau}_1={m}_1{r}_1\left[{r}_1{\ddot{\theta}}_1+{\ddot{x}}_a\mathit{\cos}{\theta}_1+{\ddot{y}}_a\mathit{\sin}{\theta}_1+ gsin{\theta}_1\right]+{m}_2{L}_1\left[{L}_1{\ddot{\theta}}_1+{\ddot{x}}_a\mathit{\cos}{\theta}_1+{\ddot{y}}_a\mathit{\sin}{\theta}_1+ gsin{\theta}_1\right]+{m}_2{r}_2\left[-{r}_2{\ddot{\theta}}_2+ gsin{\theta}_2+{\ddot{x}}_a\mathit{\cos}{\theta}_2+{\ddot{y}}_a\mathit{\sin}{\theta}_2+{L}_1\left({\ddot{\theta}}_1+{\ddot{\theta}}_2\right)\cos \left({\theta}_1-{\theta}_2\right)+{L}_1\left({\dot{\theta}}_1^2+{\ddot{\theta}}_2\right)\sin \left({\theta}_1-{\theta}_2\right)\right]+{I}_2{\ddot{\theta}}_2+{I}_1{\ddot{\theta}}_1-{L}_2{F}_1\mathit{\cos}{\theta}_2-{L}_1{F}_1\mathit{\cos}{\theta}_1-{L}_2{F}_2\mathit{\sin}{\theta}_2-{L}_1{F}_2\mathit{\sin}{\theta}_1 $$

22 $$ {\tau}_2={m}_2{r}_2\left[{r}_2{\ddot{\theta}}_2+{\ddot{x}}_a\mathit{\cos}{\theta}_2+{\ddot{y}}_a\mathit{\sin}{\theta}_2+ gsin{\theta}_2+{L}_1{\ddot{\theta}}_1\cos \left({\theta}_1-{\theta}_2\right)-{L}_1{\dot{\theta}}_1^2\sin \left({\theta}_1+{\theta}_2\right)\right]+{I}_2{\ddot{\theta}}_2-{L}_2{F}_1\mathit{\cos}{\theta}_2-{L}_2{F}_2\mathit{\sin}{\theta}_2 $$

### Human gait analysis

The performance parameters of a prosthetic leg can be judged on the basis of how well it mimics the normal human leg. For that purpose, robotic-leg gait patterns can be compared with those of humans taken as a reference. In other words, rehabilitation effectiveness can be measured based on how precisely the amputee can reproduce the kinematics of a healthy person. For modeling purposes, kinematic parameters obtained through gait analysis are necessary.

**Fig. 7:**
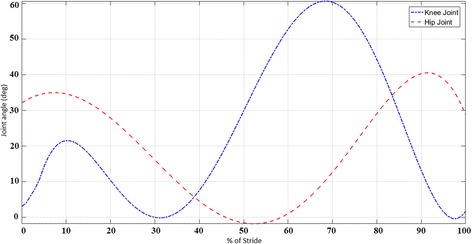
Joint angles in lower extremities during walking

Uniformity in hip, knee and ankle joint angles has been noted in further analyses of gait cycles at selected walking paces [71, 72]. Fig. 7 represents the mean joint angles for one complete stride. As there is no major variation from person to person, the mean values can be used as a standard for the input joint-angle trajectory [72, 73].

### Control strategy

The selected joints torque requires effective control in order to synchronize it with the natural joint-angle trajectory [62, 74–77]. To mimic the natural leg, prosthetic-leg position-error minimization by the proportional derivative computed torque controller (PD-CTC) with gravity compensation has been proposed [40, 78].

This is also known as inverse dynamic control, in which the system is cascaded with its inverse to take the overall system gain to unity. Usually, the inverse is incorporated with errors, and so a feedback loop is added for compensation [40, 59, 79].

The computed torque controller (CTC) is given by

23 $$ Q=M(q)\left\{{K}_v\left({\ddot{q}}^{\ast }+{\dot{q}}^{\ast }+\dot{q}\right)+{K}_p\left({q}^{\ast }-q\right)\right\}+b\left(q,\dot{q}\right)+F\left(\dot{q}\right)+g(q) $$

24 $$ =D\left(q,\dot{q},\left({\ddot{q}}^{\ast }+{K}_v\Big({\dot{q}}^{\ast }-\dot{q}\right)+{K}_p\left({q}^{\ast }-q\right)\right)\Big) $$

where *K*_*v*_ and *K*_*p*_ are damping matrices or velocity and position gains, and *D*(.) is the inverse dynamics function.

The inverse dynamics are evaluated at each servo interval. However, the coefficients matrices *M*, *b* and *g* can be evaluated at a lower rate, as the manipulator configuration changes relatively slowly. Assuming ideal parameterization, the error dynamics of the system are modeled as

25 $$ \ddot{e}+{K}_v\dot{e}+{K}_pe=0 $$

where *e* = *q*^∗^ − *q*. The joint errors are uncoupled; therefore, their dynamics are independent of manipulator configuration.

**Fig. 8:**
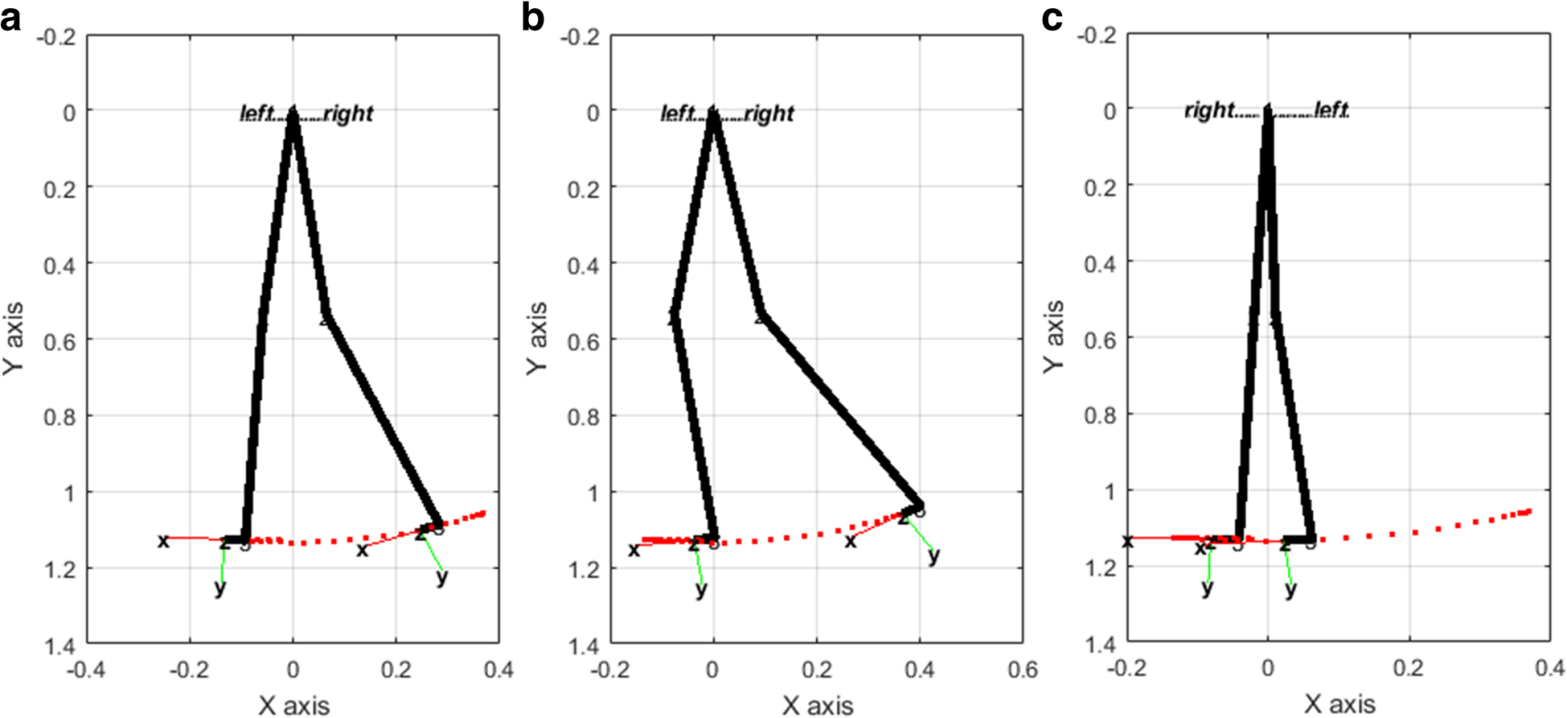
Side view of biped robot at mid stance (**a**), terminal stance (**b**) and mid swing (**c**)

In the present study, prosthetic leg simulations were performed with different stride lengths given by the National Center for Health Statistics [65, 66]. Figure 8 shows a simulation plot of the biped robot at different instants.

**Fig. 9:**
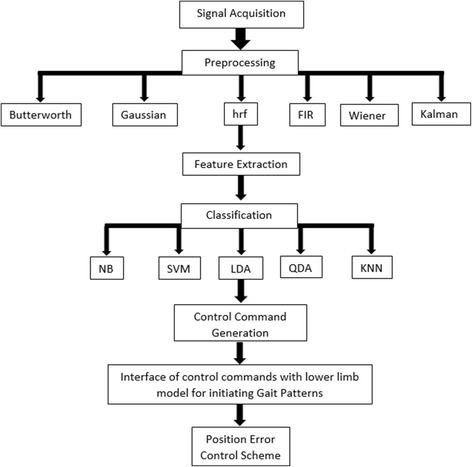
Processing pipeline of the complete system

The complete processing pipeline of entire methodology from signal acquisition to control scheme for minimization of position error is given in Fig. 9. After signal acquisition signals are preprocessed using six filters. Then six statistical features are spatially extracted across 12 channels. Later this data is classified using five different classifiers for comparative analysis of accuracies. Afterwards control commands based on brain intention were generated to move biped robot according to desired gait patterns with minimization of position error.

## Results

As discussed earlier, in order to achieve optimal accuracy, we compared six filters and five classifiers. The classification accuracies were obtained for two-, three- and six-extracted-feature combinations using *∆c*_HbO_(*t*) against all filters and classifiers for the nine subjects. The classification accuracies for the two- and three-feature combinations are shown in Tables 3 and 4, while the six-feature-combination classification accuracies for 6 filters are shown in Table 5. After analyzing Table 5, it was observed that using FIR, Gaussian, Kalman, Wiener and Butterworth processed signals accuracies were below acceptable benchmark for BCI [21]. Moreover, consistent best accuracies were 77.5, 72.5, 68.3, 74.2, 73.3, 80.8, 65, 76.7, and 86.7% for the nine subjects, respectively, as obtained using the SVM classifier with hrf processed signals. The statistically significant *p*-values of the classifiers for the HbO signals shown in Table 6 verify the greater statistical significance of the SVM over all of the other above-noted classifiers. The confidence interval was adjusted to 0.01 after applying Bonferroni correction of multiple comparisons. The results also demonstrate the significant effect of selection of filtering technique on classification accuracies. The below acceptable benchmark accuracies obtained using FIR, Butterworth, Kalman, Gaussian and Wiener filters, for this specific task, does not imply their futility for BCI studies. These filters have been shown to work well for several other tasks, for example, motor imagery, mental arithmetic etc. in previous studies [20, 23, 29, 47, 55, 80–84].

**Table 3:** Classification accuracies of 9-subjects across 6-filters using 2-feature combination for 6-classifiers

Feature	Accuracy (%)														
	S1/S2/S3					S4/S5/S6					S7/S8/S9				
	KNN	LDA	QDA	NB	SVM	KNN	LDA	QDA	NB	SVM	KNN	LDA	QDA	NB	SVM
SP, SK	56.7	49.2	51.7	62.5	41.7	64.6	56.3	62.1	64.6	62.5	60.4	60.4	69.2	66.3	58.3
SM, SK	57.5	52.1	57.1	62.9	59.2	62.5	66.3	62.1	66.3	65.0	60.4	60.8	60.8	63.8	61.7
SS, SK	57.1	51.7	62.9	66.7	66.7	60.8	57.9	59.2	61.3	52.5	60.8	57.9	60.0	62.5	57.5
KR, SK	57.9	54.6	41.3	62.1	55.0	63.3	61.7	57.9	62.5	63.3	67.1	60.4	60.4	61.7	52.5
VR, SK	57.1	48.3	50.0	62.5	47.5	60.0	53.3	63.3	67.5	62.5	60.8	61.3	67.9	65.0	63.3
SP, KR	52.1	45.4	54.2	62.1	54.2	54.2	66.3	60.0	62.5	74.2	65.0	58.3	61.3	64.2	60.0
SM, KR	52.5	57.1	62.1	63.8	59.2	54.6	68.3	63.3	63.3	70.8	65.0	58.8	59.2	63.8	55.0
SS, KR	52.9	54.6	65.8	67.5	68.3	55.4	63.3	55.4	64.2	68.3	65.0	58.3	63.3	62.9	59.2
VR, KR	52.9	42.5	47.1	62.1	53.3	55.8	62.5	63.8	67.5	74.2	65.0	65.4	62.5	64.6	65.0
SM, SP	57.9	52.1	59.2	62.5	51.7	63.8	64.2	59.2	62.9	72.5	57.9	60.4	60.0	62.9	57.5
SM, VR	61.3	51.7	54.2	61.3	55.8	61.3	60.8	63.8	69.6	71.7	57.1	60.0	59.2	65.0	60.0
SS, SP	60.0	55.4	67.9	66.7	70.0	65.0	43.3	56.3	60.4	62.5	58.8	61.7	62.9	65.0	58.3
SS, VR	57.1	52.9	63.3	66.3	72.5	62.9	40.8	63.8	70.4	65.8	62.1	65.4	64.2	64.6	60.0
VR, SP	52.5	51.3	51.7	62.5	40.0	59.2	50.0	61.3	63.3	67.5	49.6	60.8	67.1	65.0	62.5
SM, SS	61.3	54.6	64.6	68.3	66.7	72.5	56.7	53.8	59.2	61.7	58.8	60.8	59.6	63.3	60.0
SP, SK	58.3	51.7	62.5	63.3	59.2	54.2	47.9	52.1	63.8	65.0	52.5	43.3	40.8	60.8	48.3
SM, SK	58.3	55.4	64.2	67.1	69.2	56.3	45.4	56.3	64.2	60.8	50.0	50.8	46.3	59.2	61.7
SS, SK	57.9	64.2	55.4	68.3	70.8	57.1	62.5	58.8	62.5	64.2	51.3	66.3	65.8	62.9	70.0
KR, SK	67.1	63.3	71.3	62.9	67.5	60.0	57.1	57.1	61.7	55.8	51.3	57.1	58.3	62.1	59.2
VR, SK	57.9	51.3	61.3	63.8	60.8	56.7	50.4	55.4	60.0	63.3	51.7	54.6	58.8	62.1	65.0
SP, KR	55.4	53.3	62.9	62.5	57.5	55.0	50.4	56.7	62.1	55.8	52.1	55.0	59.2	60.8	59.2
SM, KR	54.2	57.5	62.9	68.3	65.8	55.0	51.7	59.6	60.0	63.3	52.1	56.3	56.7	60.8	58.3
SS, KR	54.2	62.1	48.8	63.3	65.8	53.3	59.6	65.0	60.4	69.2	52.9	71.3	68.3	62.1	68.3
VR, KR	54.2	43.8	51.7	61.3	53.3	52.9	56.3	51.3	60.8	59.2	52.9	56.7	62.9	62.1	65.0
SM, SP	67.9	56.7	72.9	64.6	66.7	64.2	42.9	58.8	63.3	61.7	60.8	47.9	59.6	59.2	53.3
SM, VR	65.8	55.8	72.1	65.0	68.3	59.6	44.6	64.2	61.7	65.8	55.4	47.9	62.1	62.9	65.0
SS, SP	53.8	62.1	56.7	64.2	61.7	69.2	57.9	59.2	64.6	63.3	66.7	67.5	67.9	62.5	62.5
SS, VR	59.6	61.7	55.4	62.9	61.7	57.5	62.1	60.8	63.3	64.2	59.6	66.3	67.1	64.6	65.0
VR, SP	49.2	55.0	64.6	62.1	59.2	59.6	53.3	60.8	60.8	62.5	54.6	55.0	65.0	62.9	69.2
SM, SS	60.8	62.9	63.8	66.7	65.8	75.0	58.3	59.2	67.1	67.5	75.0	68.3	68.8	63.3	75.8
SP, SK	59.6	58.8	59.6	61.3	59.2	47.1	57.5	55.8	55.8	52.5	64.2	63.8	71.3	70.4	76.7
SM, SK	59.6	62.1	62.9	64.6	59.2	47.5	53.8	51.7	57.9	50.8	65.8	65.8	72.5	72.9	76.7
SS, SK	59.6	56.7	59.6	63.8	63.3	47.5	55.4	57.1	63.8	63.3	65.8	67.1	73.8	75.8	80.0
KR, SK	62.1	48.3	49.6	62.5	51.7	54.2	52.5	46.3	62.5	50.0	77.5	67.5	65.8	72.1	78.3
VR, SK	59.6	59.6	57.1	60.0	60.8	47.5	60.8	58.8	62.9	55.8	65.8	63.8	75.0	70.4	75.8
SP, KR	56.7	39.6	54.2	62.5	56.7	57.9	58.8	57.9	59.2	56.7	71.3	67.5	74.2	69.2	74.2
SM, KR	55.8	43.8	60.4	60.0	58.3	58.8	53.3	53.8	62.5	55.8	71.3	66.3	74.2	72.9	80.0
SS, KR	55.4	44.2	49.2	62.5	50.0	58.3	56.3	63.8	65.0	62.5	71.3	71.7	73.8	71.3	79.2
VR, KR	55.4	50.8	47.5	51.7	50.8	58.3	60.8	65.4	62.9	54.2	71.3	66.7	73.8	68.8	72.5
SM, SP	57.1	38.3	57.1	58.3	57.5	57.1	55.8	57.1	59.6	53.3	73.8	59.6	67.5	62.1	70.8
SM, VR	64.2	49.2	63.8	56.7	57.5	60.4	58.8	60.0	64.6	57.5	61.7	56.3	63.8	62.1	70.8
SS, SP	52.5	41.7	47.9	61.3	57.5	65.8	62.1	63.3	65.8	63.3	66.3	60.4	72.5	70.8	74.2
SS, VR	62.5	52.1	54.2	52.5	55.8	65.4	63.8	62.1	65.8	70.8	66.3	57.9	68.8	73.3	70.0
VR, SP	54.6	57.5	55.0	48.3	57.5	53.3	62.5	65.0	62.5	52.5	57.9	62.9	74.6	65.4	75.8
SM, SS	66.7	47.5	61.3	58.3	57.5	73.8	57.1	54.6	65.0	63.3	77.5	60.4	77.1	78.8	85.8

**Table 4:** Classification accuracies of 9-subjects across 6-filters using 3-feature combination for 6-classifiers

Feature	Accuracy (%)														
	S1/S2/S3					S4/S5/S6					S7/S8/S9				
	KNN	LDA	QDA	NB	SVM	KNN	LDA	QDA	NB	SVM	KNN	LDA	QDA	NB	SVM
SM, SP, SS	60.4	58.8	71.7	67.5	74.2	70.8	63.8	58.3	63.3	73.3	58.8	61.7	65.8	63.3	60.0
SM, SP, KR	52.5	52.9	60.0	62.1	65.8	54.2	68.8	64.2	63.3	75.0	65.0	58.8	59.2	65.4	51.7
SM, SP, SK	56.7	54.6	55.4	61.3	58.3	64.6	65.8	63.8	62.1	69.2	60.4	59.2	63.8	62.9	60.8
SM, SP, VR	57.9	50.0	57.1	57.5	60.8	63.8	66.7	66.3	65.8	73.3	57.9	59.6	62.5	62.9	56.7
SS, SP, SK	56.7	55.4	57.5	66.7	75.0	64.6	55.8	62.9	64.6	64.2	60.4	62.1	63.3	67.1	64.2
SP, KR, VR	52.1	52.5	56.7	60.4	55.8	54.2	67.1	62.9	66.3	75.8	65.0	64.6	66.7	67.5	63.3
SP, SS, VR	60.0	55.8	61.3	66.3	72.5	65.0	44.6	58.3	64.6	71.7	58.8	60.4	66.3	64.2	57.5
VR, SS, KR	52.9	50.4	60.8	67.5	69.2	55.4	62.1	60.4	66.3	77.5	65.0	64.6	65.0	66.7	63.3
VR, SS, SM	61.7	60.8	69.2	66.3	73.3	72.5	58.3	64.6	70.0	71.7	58.8	62.9	60.8	64.6	62.5
VR, SM, SK	57.5	53.3	54.2	56.7	60.0	62.5	65.8	66.7	67.5	68.3	60.4	57.9	67.5	65.4	61.7
VR, SM, KR	52.5	52.5	56.3	58.8	60.8	54.6	67.5	67.1	70.0	80.0	65.0	60.8	62.9	67.1	57.5
KR, SP, SS	52.1	54.2	65.0	67.5	68.3	54.2	65.8	59.6	62.5	73.3	65.0	61.3	63.3	63.3	61.7
KR, SM, SK	57.9	52.1	59.6	61.7	65.0	63.3	67.9	63.3	64.2	69.2	67.1	63.3	59.2	62.5	55.0
SS, SM, SK	57.5	55.4	64.6	68.3	67.5	63.3	65.8	61.3	65.0	63.3	60.4	59.6	61.7	63.3	60.8
SS, SK, KR	57.9	56.7	48.8	66.7	73.3	63.3	61.3	57.5	62.5	70.0	67.1	58.8	64.2	59.6	56.7
SS, SM, KR	52.5	55.8	68.8	67.1	68.3	54.6	68.3	63.8	64.2	67.5	65.0	64.6	65.4	66.3	59.2
SS, SK, VR	57.1	55.8	54.6	68.3	75.0	60.8	54.2	62.1	68.8	62.5	60.8	63.3	66.3	65.0	69.2
SK, VR, KR	57.9	51.3	47.1	60.0	49.2	63.3	62.1	58.8	64.6	68.3	67.1	64.2	65.0	64.6	68.3
SK, SP, KR	57.9	50.8	46.7	61.3	48.3	63.3	67.1	62.1	62.1	71.7	67.1	60.4	65.4	63.3	55.8
SK, VR, SP	56.7	50.8	54.2	55.8	45.8	64.6	56.7	62.5	67.9	70.8	60.4	62.1	64.2	67.1	59.2
SM, SP, SS	70.4	57.5	73.8	65.4	74.2	81.3	57.9	60.4	62.9	70.8	73.3	67.5	70.8	61.7	72.5
SM, SP, KR	55.8	55.0	70.8	65.8	71.7	56.7	50.8	65.8	62.1	63.3	53.3	60.0	59.6	59.2	64.2
SM, SP, SK	58.3	53.8	70.0	66.3	70.0	54.2	44.2	61.3	62.5	62.5	51.3	48.3	53.8	57.9	50.8
SM, SP, VR	67.9	54.2	72.5	62.1	76.7	64.2	47.1	62.9	60.0	65.0	60.8	54.6	58.8	61.3	66.7
SS, SP, SK	58.3	62.1	56.3	67.9	70.0	54.2	59.2	59.2	62.5	65.8	52.5	65.4	65.0	60.8	72.5
SP, KR, VR	55.4	54.2	62.5	60.8	60.0	55.0	53.3	57.1	60.4	55.8	52.1	57.9	62.5	61.7	62.5
SP, SS, VR	53.8	60.0	62.9	65.0	69.2	69.2	57.9	63.8	64.6	63.3	66.7	69.2	70.8	62.5	70.0
VR, SS, KR	54.2	60.8	50.0	65.8	64.2	53.3	59.2	58.8	61.3	68.3	52.9	72.5	68.8	65.4	67.5
VR, SS, SM	61.7	57.9	69.6	64.6	72.5	75.0	59.6	65.4	65.0	73.3	75.0	67.1	72.5	63.3	69.2
VR, SM, SK	58.3	54.6	70.4	65.4	71.7	56.3	46.3	62.5	60.0	65.8	50.0	49.6	60.8	60.8	65.8
VR, SM, KR	54.2	53.8	72.1	66.3	68.3	55.0	54.6	66.7	58.3	66.7	52.1	59.2	65.0	61.3	68.3
KR, SP, SS	55.4	60.8	51.3	63.8	64.2	54.6	55.4	57.9	62.9	68.3	52.1	70.4	70.0	60.4	70.8
KR, SM, SK	67.1	62.9	75.0	66.3	70.8	60.0	53.3	57.1	61.3	57.5	51.3	55.0	54.6	60.4	60.8
SS, SM, SK	58.3	62.1	63.8	70.0	70.0	56.3	59.6	59.2	64.2	67.5	50.0	68.3	70.0	62.9	73.3
SS, SK, KR	67.1	61.7	66.7	69.2	76.7	60.0	59.6	63.8	58.8	68.3	51.3	72.5	70.0	62.5	69.2
SS, SM, KR	54.2	60.0	59.2	65.8	70.0	55.0	59.2	63.8	61.3	70.8	52.1	69.2	71.7	63.8	78.3
SS, SK, VR	57.9	62.1	56.3	69.2	72.5	57.1	62.9	59.6	61.3	68.3	51.3	66.3	65.0	64.2	62.5
SK, VR, KR	67.1	57.1	73.3	62.9	68.3	60.0	55.4	56.7	59.6	60.0	51.3	54.2	61.3	62.1	67.5
SK, SP, KR	67.1	58.8	74.6	62.5	70.0	60.0	50.0	54.2	61.7	55.0	51.7	55.4	57.9	61.3	53.3
SK, VR, SP	58.3	51.7	56.7	60.4	59.2	54.2	53.3	61.7	61.3	61.7	52.5	51.3	61.7	61.3	60.8
SM, SP, SS	61.7	43.8	57.1	55.8	55.8	68.8	60.4	71.7	62.5	75.0	80.4	60.8	76.3	76.3	83.3
SM, SP, KR	56.7	38.8	56.3	56.7	61.7	57.5	56.3	57.9	56.3	55.0	71.3	65.4	65.4	74.6	80.0
SM, SP, SK	59.6	60.4	64.2	59.6	60.0	47.1	57.1	58.3	57.1	54.2	64.2	62.5	66.7	72.9	81.7
SM, SP, VR	57.1	62.9	64.2	55.0	58.3	57.1	62.1	64.6	62.1	53.3	73.8	62.9	63.8	63.8	77.5
SS, SP, SK	59.6	60.4	59.6	60.4	58.3	47.1	63.8	64.6	64.6	63.3	64.2	63.8	73.3	76.3	79.2
SP, KR, VR	56.7	56.3	56.3	49.6	59.2	57.9	62.9	65.0	62.1	53.3	71.3	65.8	69.6	67.9	77.5
SP, SS, VR	52.5	57.1	55.4	47.5	55.0	65.8	64.6	67.9	63.8	65.0	66.3	62.5	76.3	72.5	82.5
VR, SS, KR	55.4	56.7	53.8	51.3	58.3	58.3	62.5	67.5	65.4	80.0	71.3	66.3	72.1	70.8	77.5
VR, SS, SM	66.7	50.8	64.6	55.8	58.3	74.2	62.9	66.3	62.9	68.3	77.9	60.4	73.3	75.4	83.3
VR, SM, SK	59.6	62.1	65.4	58.8	60.8	47.5	58.8	58.8	62.1	56.7	65.8	63.3	67.9	72.1	82.5
VR, SM, KR	55.8	47.5	61.7	55.4	60.8	58.8	63.3	62.5	62.1	57.5	71.3	65.4	65.8	68.3	81.7
KR, SP, SS	56.7	42.1	54.2	60.4	58.3	57.9	60.4	65.4	66.7	69.2	71.3	67.1	72.1	72.9	80.8
KR, SM, SK	62.1	62.5	60.8	62.9	64.2	54.2	53.8	57.1	55.8	55.0	77.5	66.3	70.4	74.6	75.8
SS, SM, SK	59.6	60.0	66.7	59.6	60.8	47.5	57.1	51.7	61.7	65.0	65.8	66.3	68.3	78.3	85.8
SS, SK, KR	62.1	55.0	55.0	63.8	48.3	54.2	55.0	57.9	65.0	67.5	77.5	71.3	73.8	73.8	80.8
SS, SM, KR	55.8	46.7	62.9	58.3	56.7	58.8	56.7	64.6	64.2	65.0	71.3	70.8	70.4	77.1	86.7
SS, SK, VR	59.6	58.8	59.2	58.8	58.3	47.5	64.2	68.3	63.8	64.2	65.8	64.2	74.2	74.2	80.0
SK, VR, KR	62.1	59.2	58.3	58.3	64.2	54.2	60.4	65.0	61.3	60.0	77.5	68.8	70.8	70.4	77.5
SK, SP, KR	62.1	60.0	59.2	63.8	62.5	54.2	59.2	60.0	50.0	58.3	77.5	67.9	72.9	73.3	75.8
SK, VR, SP	59.6	57.5	53.3	57.1	60.8	47.1	59.6	62.9	62.1	52.5	64.2	60.4	67.9	71.3	80.0

**Table 5:** Classification accuracies of 9-subjects across 6-filters using 6-feature combination for 6-classifiers

Filter	Accuracy (%)														
	S1 S2 S3					S4 S5 S6					S7 S8 S9				
	KNN	LDA	QDA	NB	SVM	KNN	LDA	QDA	NB	SVM	KNN	LDA	QDA	NB	SVM
FIR	56.7	60.8	61.3	64.6	55.0	55.0	59.2	66.3	62.9	59.2	59.6	57.5	60.0	59.6	64.2
	65.0	50.4	67.9	57.5	63.3	56.7	62.1	62.5	62.5	58.3	50.4	61.7	63.3	63.8	53.3
	59.2	60.8	52.1	53.8	62.5	61.7	60.0	58.3	52.5	50.0	56.7	69.2	74.2	71.3	67.5
hrf	57.9	62.2	69.2	68.3	77.5	63.3	65.8	63.3	64.2	74.2	67.1	61.3	64.6	65.0	65.0
	67.1	60.4	75.8	70.8	72.5	60.0	61.2	67.1	60.8	73.3	51.7	72.1	72.9	62.9	76.7
	62.1	63.8	67.5	55.8	68.3	54.2	60.4	71.7	59.2	80.8	77.5	68.8	80.0	73.3	86.7
Gaussian	56.7	59.2	53.8	62.5	65.0	54.2	52.1	55.0	57.9	55.8	57.1	53.8	48.3	57.5	54.2
	50.0	55.8	52.5	61.7	58.3	52.1	50.0	52.1	58.8	60.0	55.8	55.0	53.3	60.4	55.0
	52.1	59.6	50.8	54.2	57.5	55.4	60.0	64.2	57.1	54.2	48.8	57.5	59.2	61.7	61.7
Kalman	51.3	57.1	52.5	64.2	65.0	53.8	53.3	52.9	54.2	51.7	47.1	56.7	55.0	57.9	60.8
	53.3	46.7	55.0	60.4	54.2	52.5	48.8	49.6	59.2	47.5	51.3	60.0	57.1	60.0	51.7
	55.4	57.9	47.9	52.1	55.0	60.4	57.1	62.5	57.5	50.0	56.7	57.5	64.6	61.3	57.5
Wiener	60.0	52.9	57.9	63.8	65.8	57.9	55.4	60.0	57.1	65.0	55.4	57.5	63.3	62.9	70.8
	59.2	62.5	70.8	63.3	68.3	51.3	55.8	59.6	56.7	70.0	54.6	62.9	65.0	64.6	72.5
	52.9	59.6	56.7	58.8	65.0	55.0	57.5	59.6	63.3	60.8	62.1	60.4	70.0	70.0	78.3
Butterworth	55.4	60.8	60.0	62.9	65.0	49.2	55.4	63.3	59.6	53.3	56.3	56.7	60.4	62.5	55.8
	60.0	57.5	67.1	65.0	63.3	54.2	55.4	64.2	59.6	65.0	52.9	60.0	61.7	62.9	63.3
	48.8	59.2	56.3	52.1	57.5	43.8	60.0	64.2	67.1	55.8	62.1	63.8	63.8	60.8	63.3

**Table 6:** Statistical significances of classifiers

Classifiers	*p-*values
KNN vs. LDA/QDA/NB/SVM	0.158, 0.016, 0.158, 0.009
LDA vs. QDA/NB/SVM	0.009, 0.369, 0.001
QDA vs. NB/SVM	0.047, 0.009
NB vs. SVM	0.002

### Online BCI

In online BCI, we require minimum computation so as to reduce execution delay for control-command generation. Most of the previous fNIRS-BCI studies have used LDA for online classification, because it provides a balance between time of execution and classification accuracy [23]. Thus, in our study, we used LDA with six-feature combinations. For real-time BCI, we divided the total of 10 trials into two sections: one section of 9 training trials and the other of 1 testing trial. The classifier was first trained offline using 9 trials having 10 runs with ten-fold cross-validation. The one-time-trained classifier was then used to classify the one unknown testing trial in online BCI. To avoid a false trigger of a control command, the testing trial was randomly divided into 10 indices having observations approximately equal to 10 disjoint subsets. Each subset was then classified to make a binary decision. Based on the ten-fold classified data, an average threshold of “90% true” was set for accurate triggering.

### Error plots

**Fig. 10:**
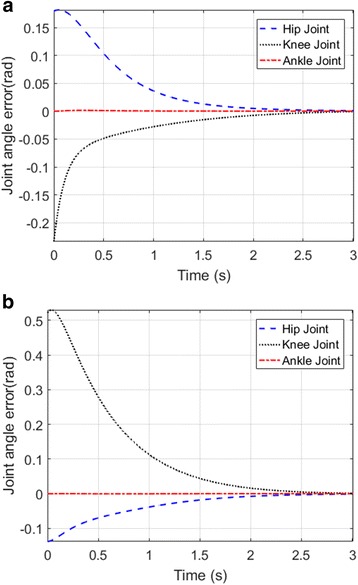
Joint-angle error plot of left leg (**a**) and right leg (**b**)

**Fig. 11:**
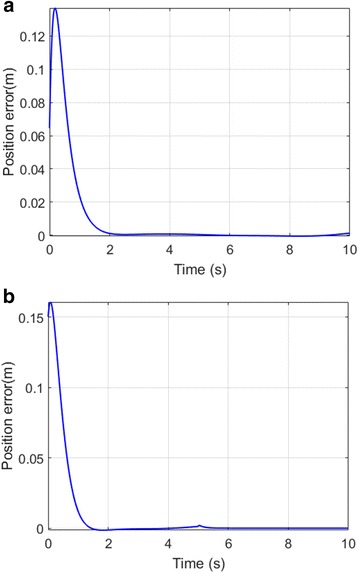
Tool tip position-error plot of left leg (**a**) and right leg (**b**)

The trigger command generated based on brain intention is used to generate gait cycles of a prosthetic leg through given human joint-angle trajectories, while the PD-CTC controller minimizes joint angle and position error. Joint-angle and position-error plots for reference input trajectories of the left and right leg are provided in Figs. 10 and 11, respectively.

**Fig. 12:**
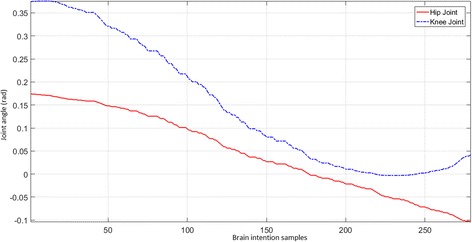
Brain intention versus joint angles for Subject 1

Figure 12 provides a brain intention versus joint-angle plot. When the rest intention is transmitted, the prosthetic leg retains its previous joint angles while updating the next input joint angles for the walk intention.

## Discussion

In past studies, researchers have endeavored to improve classification accuracies by using different feature combinations or by making changes to machine-learning algorithms. The frequently used features are signal mean (SM) [20, 29, 48, 83, 85–88], signal slope (SS) [20, 29, 52, 83, 89], signal variance (SV) [52, 86], slope kurtosis (KR) [86], signal peak (SP) [52, 86, 90] and signal skewness (SK) [52, 86]. To avoid false triggers in rehabilitation, the consistently best accuracies achieved using six- dimensional feature combinations have been considered compulsory, as reported in [55]; however, in the present study, for the 2-feature combination SM/SS, the best average accuracy, 67%, was achieved, while for the three-feature combination SM/SP/SS, an optimal average accuracy of 71% was obtained. Similar 2-feature combinations have been reported for two-class imagery movement by Naseer et al. [29], who, using time windows of SS and SM for right- and left-wrist motor imageries, increased accuracies from 83 to 87.28%. Due to individual-participant differences, these classification accuracies varied. The differences might have been due to scalp-cortex distance and head shape, both of which can cause major variation, as reported in [29]. The low classification accuracies might have been due to the fact that the hemodynamic responses of people with motor impairment due to tetraplegia or multiple sclerosis differ as compared with healthy persons, as discussed in [91]. Moreover, an optimal classifier also plays a vital role in enhancing performance accuracies, as reported in [55], where five classifiers were compared to obtain the maximum accuracy. For the present study, the proposed classifiers were LDA and SVM, as also reported in [23, 48, 49, 53, 55, 92].

For an online interface, we proposed a novel methodology in which the testing trial is divided into 10 indices and each subsection is separately classified. The triggering command was generated based on a 90% average true benchmark of classified subsections. A similar real-time interface was reported in [23], but it used a separate framework for binary decoding. Furthermore, for a normal walk task reported in [22] based on the use of an online interface, a separate methodology also was used, in that case to synchronize the triggers of fNIRS signals and the gait system.

In the second part of this study a proposed nonlinear position control for a prosthetic leg was studied for gait-rehabilitation purposes. An independent, self-sufficient mechanism was developed that can mimic the normal human gait pattern based on PD-CTC. It is evident from Fig. 9 that the controller minimized the position error in less than 2.5 s. The same strategy was seen in [33], which minimized joint error using a sliding-mode control, but a steady-state error was observed. Similarly, when adaptive control was applied in a previous study [32], a constant error was seen across hip movement in the reported results. Moreover, consistent error was observed across knee and ankle angles in [77], which reported that error increases with increaing torque bounds.

fNIRS is an indirect optical measurement technique that measures hemodynamic changes instead of neural activity. Accordingly, there is always a delay between an activity performed and a detected response; thus, in such decoding tasks, classification accuracy is compromised. With advanced filtering techniques [11, 93, 94], different feature combinations [81] and various classification techniques [55, 80], accuracies can be increased. One additional limitation of this study is that it generates the control command based on the walk intention whereas during the rest intention it holds the lower limb to its last updated position. In order to return the lower limb to its initial state with the rest intention, a methodology that incurs shorter computation and execution time needs to be developed.

## Conclusion

The aims of this study were to use an optimal filter and classifier to obtain the maximum accuracy for given data and to implement a gait control scheme for a lower limb. To those ends, fNIRS signals were acquired from the primary motor cortex (M1) in the left hemisphere of the brain. For removal of physiological and instrumental noises, six filters (i.e. Kalman, Wiener, Gaussian, hemodynamic response (hrf), Band-pass, finite impulse response (FIR)) were used with the five classifiers QDA, LDA, SVM, KNN and NB. Brain intention was used to generate trigger commands, while the computed torque controller (CTC) was used to reduce position error. For brain-signal classification, six-feature (i.e. SS, SP, SM, KR, SV, SK) combinations were used. An average accuracy of 75% was obtained using the SVM offline classifier with hrf. For rehabilitation purposes, online classification was performed using LDA. To avoid false triggering, the testing trial was divided into 10 further subsections, and each subsection was separately classified. The triggering command was generated based on a 90% average accuracy benchmark for classified sections. In the second part of this study, a proposed prosthetic leg model was derived that is non-linear in nature; thus, it was determined that the nonlinear characteristics of the system could not be ignored. Therefore, instead of applying linearization to solve this problem approximately, we utilized the PD-CTC with guaranteed global asymptotic stability. The proposed prosthetic leg model was more deeply explored using the Euler Lagrange approach. A simple PD-CTC independent joint controller was utilized for the hip and knee joints so that the manipulator retained its nonlinear characteristics. The simulation results confirmed that the asymptotic stability of the system can be reached in a finite time, as the determined position accuracy was satisfactory. Possible extension of this work would entail increasing the number of BCI classes for exploration of the gait patterns of persons of different age groups. Another interesting aspect could be exploring the relevance of individual channels with the task. Using features from more relevant channels for classification might also increase the classification accuracy.
